# Adult and Cord Blood-Derived High-Affinity gB-CAR-T Cells Effectively React Against Human Cytomegalovirus Infections

**DOI:** 10.1089/hum.2019.149

**Published:** 2020-04-16

**Authors:** Henning Olbrich, Sebastian J. Theobald, Constanze Slabik, Laura Gerasch, Andreas Schneider, Michael Mach, Thomas Shum, Maksim Mamonkin, Renata Stripecke

**Affiliations:** ^1^ Laboratory of Regenerative Immune Therapies Applied, Department of Hematology, Hemostasis, Oncology and Stem Cell Transplantation, Hannover Medical School, Hannover, Germany.; ^2^ German Center for Infection Research (DZIF), Partner Site Hannover-Braunschweig, Braunschweig, Germany.; ^3^ Institute for Clinical and Molecular Virology, Friedrich-Alexander-Universität Erlangen-Nürnberg, Erlangen, Germany.; ^4^ Center for Cell and Gene Therapy, Baylor College of Medicine, Houston, Texas.; ^5^ Medical Scientist Training Program, Baylor College of Medicine, Houston, Texas.; ^6^ Department of Pathology and Immunology, Baylor College of Medicine, Houston, Texas.

**Keywords:** HCMV, CAR-T cells, transplantation, PD-1, humanized mice

## Abstract

Human cytomegalovirus (HCMV) reactivations are associated with lower overall survival after transplantations. Adoptive transfer of HCMV-reactive expanded or selected T cells can be applied as a compassionate use, but requires that the human leukocyte antigen-matched donor provides memory cells against HCMV. To overcome this, we developed engineered T cells expressing chimeric antigen receptors (CARs) targeted against the HCMV glycoprotein B (gB) expressed upon viral reactivation. Single-chain variable fragments (scFvs) derived from a human high-affinity gB-specific neutralizing monoclonal antibody (SM5-1) were fused to CARs with 4-1BB (BBL) or CD28 (28S) costimulatory domains and subcloned into retroviral vectors. CD4^+^ and CD8^+^ T cells obtained from HCMV-seronegative adult blood or cord blood (CB) transduced with the vectors efficiently expressed the gB-CARs. The specificity and potency of gB-CAR-T cells were demonstrated and compared *in vitro* using the following: 293T cells expressing gB, and with mesenchymal stem cells infected with a HCMV TB40 strain expressing *Gaussia* luciferase (HCMV/GLuc). BBL-gB-CAR-T cells generated with adult or CB demonstrated significantly higher *in vitro* activation and cytotoxicity performance than 28-gB-CAR-T cells. Nod.Rag.Gamma (NRG) mice transplanted with human CB CD34^+^ cells with long-term human immune reconstitution were used to model HCMV/GLuc infection *in vivo* by optical imaging analyses. One week after administration, response to BBL-gB-CAR-T cell therapy was observed for 5/8 mice, defined by significant reduction of the bioluminescent signal in relation to untreated controls. Response to therapy was sporadically associated with CAR detection in spleen. Thus, exploring scFv derived from the high-affinity gB-antibody SM5-1 and the 4-1BB signaling domain for CAR design enabled an *in vitro* high on-target effect and cytotoxicity and encouraging results *in vivo*. Therefore, gB-CAR-T cells can be a future clinical option for treatment of HCMV reactivations, particularly when memory T cells from the donors are not available.

## Introduction

Human cytomegalovirus (HCMV) is a ubiquitous human pathogen that latently infects a large proportion of the adult population, and, in most immunocompetent individuals, it persists by low-level reactivations and reinfections without causing clinical symptoms. Following allogeneic hematopoietic stem cell transplantation (HSCT), HCMV reactivations may lead to major clinical complications.^1–4^ Early HCMV reactivation remains associated with lower overall survival and higher nonrelapse mortality.^3^ Despite the prophylactic and preemptive use of antiviral drugs, clinical manifestations, such as interstitial pneumonia, colitis, hepatitis, retinitis, or encephalitis, can escalate to end-organ diseases with up to 50% fatality.^5,6^

The HCMV-serostatus is a criterion for donor-recipient matching, that is, if the recipient is HCMV seropositive (R^+^), a matched seropositive donor (D^+^) is preferred so that cotransplanted antigen-experienced memory lymphocytes from the donor can provide some antiviral protection until immune reconstitution of the recipient has progressed. Cord blood (CB) banks are an important source of allogeneic grafts matched through the human leukocyte antigen (HLA), particularly in HLA-heterogeneous populations. However, CB-HSCTs that lack functional memory lymphocytes bear a substantially higher risk of HCMV-related complications than allogeneic HSCT using adult HCMV seropositive stem cell grafts.^7–9^

To improve antiviral responses and consequently the outcome of HSCT with HCMV seronegative donor grafts, adoptive transfer of HCMV-reactive T cells has been performed by qualified centers worldwide. Donor lymphocyte infusions have shown efficacy against HCMV reactivations.^10^ However, the approach is limited by the need for HCMV-seropositive compatible donors, is not applicable after CB-HSCT, and is associated with graft-versus-host disease (GvHD). Banking of virus-specific T cells obtained from partially HLA-matched third-party donors expanded *in vitro* with peptides has therefore been explored, but relies on the availability of partially HLA matched donors and on the variable and unpredictable T cell expansion.^11,12^ Expansion of naive T cells from banked HLA-matched CB units with peptide-loaded antigen presenting cells has been reported, but the generation is more challenging and the expanded cells recognize atypical HCMV epitopes.^13^

Genetic transfer of antigen receptors, on the contrary, can be performed after 5–10 days of T cell manipulations and 2 weeks for quality control under standardized conditions. T cells engineered with HCMV-specific HLA-restricted T cell receptor (TCR)-coding genes were shown to recognize target cells presenting the respective epitopes endogenously.^14–16^ However, TCR-transgenic T cells recognize a single HLA-restricted epitope and the downregulation of HLA classes I and II is a key immune evasion mechanism of HCMV in infected cells^17,18^ possibly limiting the activity of HLA-restricted TCR-engineered T cells.

Chimeric antigen receptor (CAR)-T cell therapy is a breakthrough approach to cancer immunotherapy and has shown substantial benefit for patients suffering from relapsed or refractory B cell malignancies^19^ and >200 CAR-T cell clinical trials have been initiated so far.^20^ Single-chain variable fragments (scFvs) derived from antigen-reactive monoclonal antibodies (mAB) incorporated into CARs mediate signaling to the T cells to react directly against antigens on the target cell membrane. Second- or third-generation CARs contain costimulatory endodomains, such as CD28 and/or 4-1BB, that improve T cell proliferation, cytokine secretion, resistance to apoptosis, and *in vivo* persistence.^19^ Furthermore, standardized and efficient Good Manufacturing Practice-compliant protocols for CAR-T cell production are available.^21^

HCMV glycoproteins abundantly expressed on the infected cell surface membrane during lytic viral infection could be explored as targets therapeutically in patients suffering from drug-refractory HCMV reactivations using CAR-T cells. The HCMV envelope glycoprotein B (gB; UL55) is the major fusogenic protein within the HCMV-fusion complex and is expressed at high levels on the cell membrane early after HCMV infection reaching peak expression levels 72–96 h after infection.^22,23^ Here, we examined the antiviral activity of HCMV-specific CAR-T cells containing the CD28 or 4-1BB costimulatory endodomains fused to scFv derived from the SM5-1 anti-gB antibody that has high-affinity binding (K_D_ = 5.7 × 10^−11^ M) to a highly conserved, nonglycosylated, and noncontiguous domain of gB (the antigenic domain IV) that is maintained during infection in both pre- and postfusion conformations.^24–26^ We show by *in vitro* experiments that gB-CAR-T cells produced from either adult blood (AB) or CB T cells recognized and killed cells infected with HCMV. For animal studies, we used our previously reported HCMV infection model based on NRG mice transplanted with CB-CD34^+^ HSCs and infected systemically with HCMV/GLuc.^27^ Our findings provide a proof-of-principle for gB-CAR-T cell therapeutic efficacy.

## Materials and Methods

### Cell lines

MRC-5 human lung fibroblasts and human embryonic kidney (HEK)-293T cells (ATCC, Manassas, VA) were cultured at 37°C, 5% carbon dioxide in Dulbecco's modified Eagle's medium (Thermo Fisher, Waltham, MA) supplemented with 10% fetal bovine serum (FBS; HyClone, Logan, UT), 1% of a 10,000 U/mL penicillin G and 10 mg/mL streptomycin sulfate solution (P/S; Merck Millipore, Billerica, MA), and for MRC-5 cultures, in addition, 1% MEM nonessential amino acid solution in Minimum Essential Medium (Thermo Fisher). A clonal gB-expressing HEK-293T cell line was established by transduction with a lentiviral vector expressing gB, selection of gB-positive clones by fluorescence-activated cell sorting, and single-cell dilution (293T-gB).

### Procurement of primary cells and tissues from healthy donor subjects

Study protocols were approved by the Ethics Committee of Hannover Medical School and peripheral blood samples were obtained from donors after informed consent. Leukapheresis units used as cell sources for production of AB CAR-T cells (AB-CAR-T) were obtained from HCMV-seronegative healthy donors identified by registries of the Institute of Transfusion Medicine at Hannover Medical School. Umbilical cord tissue and blood were obtained from healthy mothers at term through the Clinic of Gynecology and Obstetrics at Hannover Medical School.

### CAR design and CAR-T cell production

DNA fragments encoding scFvs that incorporate the variable heavy (V_H_) and variable light (V_L_) sequences of the SM5-1 antibody^24^ in both V_H_ → V_L_ and V_L_ → V_H_ orientations (containing an interspacing linker sequence) were synthesized after codon optimization (Bio Basic, Amherst, NY). The DNA fragments (with *Pme*I and *Bam*HI sites) were inserted into the SFG retroviral vector plasmids upstream of CAR backbones containing a long (C_H_2-C_H_3) or short (C_H_3 only) IgG1 Fc spacer, CD28^28^ or 4-1BB^29^ endodomains, and the CD3 ζ-chain (28S: CD28 Short spacer; 28L: CD28 Long spacer; BBL: 4-1BB Long spacer). Retroviral vectors were produced in the 293T cell line using transient cotransfection with RD114 envelope and PegPAM plasmids.^28^ Mononuclear cells (MCs) were isolated from whole blood using Ficoll density gradient separation. For AB-CAR-T cells, MCs were activated by plate-bound CD3 (OKT3; eBioscience, Waltham, MA) and CD28 antibodies (CD28.2; BioLegend, San Diego, CA) for 2 days. For CB-CAR-T cell generation, MCs were activated using activation beads conjugated with anti-CD2/CD3/CD28 antibodies (Miltenyi Biotec, Bergisch Gladbach, Germany) in a bead-to-cell ratio of 1:2 for 3 days, both in RPMI 1640 (Lonza, Basel, Switzerland) supplemented with 10% FBS, 1% P/S, and 5 ng/mL interleukin (IL)-7 and IL-15 (Miltenyi Biotec). Activated T cells were transduced by incubation for 6 h on RetroNectin-coated plates (Takara Bio, Otsu, Japan) that had been loaded by centrifugation for 60 min at 4,500*g* with γ-retroviral vectors. After transduction, CAR-T cells generated from AB were expanded in the presence of IL-7 and IL-15. Activation beads were added in combination with IL-7 and IL-15 for expansion of CAR-T cells generated from CB. T cells were harvested after expansion 1 to 2 weeks after the transduction. All experiments were performed with CAR-T cells generated from HCMV-seronegative donors or CB.

### Generation of HCMV viral stocks and infection of MRC-5 cells

The HCMV variant TB40-BAC4-GLuc expressing the secretable GLuc^30^ was propagated on MRC-5 cells. A viral stock with known titer was used to infect an MRC-5 cell layer and, 5 days after infection, a supernatant was used to reinoculate freshly seeded MRC-5 cells. Five days after the second inoculation, cell-free supernatants were collected and then concentrated by ultracentrifugation (12,500*g*, 4 h). Viral stocks were cryopreserved in the cell culture medium and titrated on MRC-5 cells by flow cytometric detection of immediate early (IE)-1 using the mouse antibody p63-27 and fluorophore-conjugated goat anti-mouse IgG (BioLegend) after permeabilization with ice-cold methanol. MRC-5 cells were seeded and infected with HCMV/GLuc at a multiplicity of infection (MOI) of 1 overnight. Three days postinfection (dpi), the batch of infected cells was aliquoted into vials and cryopreserved. After thaw, the viability of the cells and gB-expression were verified by flow cytometry.

### Generation of umbilical cord mesenchymal stem cells

Umbilical cords were cut into pieces of 1 cm length, washed with Dulbecco's phosphate-buffered saline (PBS; Biochrom, Berlin, Germany) containing 30% P/S, and cultured on tissue culture-treated dishes in alpha-MEM (Biochrom) supplemented with 10% FBS and 1% P/S (referred to as A10 from now on) until spreading growth of adherent cells became visible. The cells were detached, passaged for up to four times, and cryopreserved. Mesenchymal stem cell (MSC) lineage was confirmed by flow cytometric analysis of CD73, CD90, and CD105a expression according to the defining criteria^31^ (data not shown).

### Cocultures of CAR-T cells with HCMV-infected MSCs and detection of dead cells

MSCs were seeded onto 96-well plates at a density of 1 × 10^4^ per well and were cultured for 12 h for the cells to adhere to the wells. An HCMV/GLuc stock was diluted for a final MOI of 0.03 and was added (in a volume of 40 μL in A10 containing 20 mM 4-[2-hydroxyethyl]-1-piperazineethanesulfonic acid [HEPES]) per well. The MSCs were spinfected at 1,000*g*, 32°C, 30 min, and incubated for additional 60 min before washing. Three days after HCMV infection, when MSCs were still highly viable (>90%), CAR-T cells were added at different ratios in fresh A10. The experiments were performed in triplicate, pooled for flow cytometric analyses. For sequential reculture and killing assays, 1 × 10^5^ MSCs were seeded per well of 12-well plates and were infected at an MOI of 0.03 HCMV/GLuc in a volume of 400 μL per well. CAR-T cells were added 3 dpi at 1 × 10^5^ per well. After 5 days of coculture, the cells were resuspended and collected by gentle pipetting. A sample from each culture was stained for CD45 and analyzed by flow cytometry with counting beads to calculate the number of recovered CD45^+^ T cells. The resuspended T cells were recultured with fresh MSCs preinfected with HCMV 3 days before, as described above, restoring the starting number of 1 × 10^5^. The remaining adherent cells were trypsinized and analyzed by flow cytometry. The reculturing was repeated five times and flow cytometry analyses of the CAR-T cells were performed only after the last iteration. The experiments were performed in triplicate with MSC targets generated with three different umbilical cords. For analyses, MSC cocultures were incubated with the viability dye FVD eFluor 450 (Thermo Fisher) according to the instructions before staining with fluorochrome-labeled mAB. FVD^+^/CD45^−^ cells were defined as dead target cells.

### Analyses of proliferating T cells

CAR-T cells were labeled with the proliferation dye CellTrace Yellow (Thermo Fisher) according to the manufacturer's instructions directly before coculture. Graded dilution of the dye upon cell division was detected by flow cytometry analyses of gated CD4^+^ or CD8^+^ T cells.

### Interferon-γ detection

Interferon (IFN)-γ was detected in cryopreserved coculture supernatants using an ELISA kit (Ready-Set-Go!; Thermo Fisher). The detection limit according to the manufacturer is 4 pg/mL.

### Detection of GLuc activity by bioluminescence analyses

The bioluminescence of secreted GLuc by HCMV-infected MSCs was measured in a plate reader (TriStar^2^; Berthold Technologies, Bad Wildbach, Germany). The unfiltered bioluminescence was measured 3 s after automatic injection of 50 μL PBS containing 0.2 μg/mL coelenterazine (Promega, Madison, WI) to 20 μL of cryopreserved coculture supernatants. Culture supernatants were diluted 10- or 100-fold with PBS in cases when the bioluminescence exceeded the linear detection range guaranteed by the manufacturer.

### Generation of humanized mice infected with HCMV

All experiments involving mice were performed in accordance with the regulations and guidelines of the animal welfare of the State of Lower Saxony (Niedersächsiches Landesamt für Verbraucherschutz und Lebensmittelsicherheit, Dezernat 33/Tierschutz). NOD.Cg-*Rag1^tm1Mom^IL-2Rγ_c_^tm1Wjl^* (NRG) mice were originally obtained from The Jackson Laboratory (JAX; Bar Harbor, ME) and bred under pathogen-free conditions. Male and female mice were used for experiments. Mice were transplanted with CB-CD34^+^ cells using previously described techniques.^32–35^ Briefly, human CD34^+^ cells were isolated after two rounds of positive selection with MACS magnetic beads (CD34 MicroBead Kit; Miltenyi Biotec). At the age of 4–6 weeks, mice were sublethally irradiated with 450 cGy using a ^137^Cs-column irradiator (Gammacell 3000 Elan; Best Theratronics, Ottawa, Canada). Four hours after irradiation, 2 × 10^5^ CD34^+^ cells from a CB donor known to produce long-term human reconstitution (>20% huCD45^+^ cells in peripheral blood after 15 weeks) were injected intravenously. Infection of humanized mice with HCMV/GLuc leading to a robust and systemic viral biodistribution was performed as previously described.^27^ In brief, for infections, mice were injected for 5 days daily subcutaneously (s.c.) with 150 ng human granulocyte-colony stimulating factor (hG-CSF) (Granocyte; Kohlpharma GmbH, Merzig, Germany) to activate human stem cells and myeloid cells. On the third day of hG-CSF administration, 1 × 10^6^ thawed MRC-5/HCMV/GLuc cells were injected intraperitoneally (i.p.)

### Therapy of HCMV-infected humanized mice with gB-CAR-T cells

Eight weeks after HCMV infection, mice were administered intravenously (i.v.) with 5 × 10^5^ BBL-gB-CAR-T cells autologous to the CD34^+^-HSCT. The BBL-gB-CAR-T cells were enriched for CAR^+^/CD3^+^ cells by fluorescence-activated cell sorting and i.v. injected into the mice (“gB-CAR” given at “Week 0”). The nontreated control (CTR) experimental arm was infected with HCMV, but did not receive T cells. Optical imaging analyses of each individual mouse were performed under narcosis as described below just before euthanasia at 1 week or 4 weeks after CAR-T cell treatment. After euthanasia, blood was collected by transcutaneous heart puncture. Bone marrow (flushed from both femora), spleen, and liver were also analyzed. Organs were dissociated into single-cell suspensions; liver leukocytes were isolated by Percoll gradient separation. Blood, spleen, and liver cell suspensions were incubated in erythrocyte lysis buffer (0.83% ammonium chloride, 20 mM HEPES [pH 7.2]) for 5 min at room temperature and stabilized with PBS. The numbers of viable cells per tissue were quantified in trypan blue exclusion assays.

### *In vivo* optical imaging

To visualize GLuc expression, coelenterazine (Promega) was solubilized in ethanol to 5 mg/mL and diluted directly before administration in PBS to 0.5 mg/mL. Under anesthesia with 1 mg ketamine and 50 μg xylazine (injected i.p. in 100 μL PBS; Sigma-Aldrich, St. Louis, MO), mice were injected i.v. with 100 μL of the coelenterazine solution (50 μg coelenterazine per mouse) and luminescence images were acquired immediately using an IVIS SpectrumCT (PerkinElmer, Waltham, MA) as previously described.^27^ The anatomical regions of interest (ROIs) were kept constant for quantified analyses of all mice.

### Quantitative real-time PCR for detection of HCMV and of CAR-T cells

Snap-frozen tissue samples were processed with a blood DNA isolation kit (Qiagen, Hilden, Germany). DNA concentrations were determined by spectrophotometry. For detection of HCMV DNA, 20 μL of the DNA samples was used for PCR analyses with the Artus^®^ CMV TM PCR kit according to the manufacturer's instructions (Qiagen). Quantitative real**-**time PCR (RT-qPCR) was performed and analyzed with a StepOnePlus-PCR cycler (Thermo Fisher). C_T_ values were used to calculate the number of copies of HCMV genomes by a standard dilution curve provided with the kit and adjusted to the respective DNA concentrations. For detection of BBL-gB-CAR, 7.5 μL of the DNA samples were used for PCR analyses with TaqMan Universal Mastermix II (Applied Biosciences, Vilnius, Lithuania), 900 nM each forward and reverse primers, and 250 nM DNA probe with 5′-FAM/3′-TAM label (Eurofins, Ebersberg, Germany; sequences: forward: 5′-AGCTGCCGATTTCCAGAAGA-3′; reverse: 5′-GCGCTCCTGCTGAACTTCA-3′; Probe: [FAM] 5′-AAGGAGGATGTGAACTGAGA-3′ [TAM]). RT-qPCR was performed as described above. A standard dilution curve was measured using the retroviral plasmid DNA.

### Confocal microscopy

MSCs, 2 × 10^5^, were seeded per well on a six-well plate with a glass coverslip lying on the bottom of each well. MSCs were infected the next day as described, at an MOI of 1. Three dpi, the cells were fixed with 5% formaldehyde (15 min, room temperature), blocked with 5% FBS +0.2% Triton (Roth, Karlsruhe, Germany; 1 h, room temperature), incubated with the anti-gB mouse antibody p27-287 (overnight, 4°C), incubated with AF488-conjugated goat anti-mouse IgG (BioLegend; 1 h, room temperature), and mounted on glass slides with mounting medium containing 4′,6-diamidino-2-phenylindole (DAPI) (Vectashield; Vector Laboratories, Burlingame, CA). Images were acquired using a Leica DM IRB with a TCS SP2 AOBS scan head (Leica Microsystems, Wetzlar, Germany).

### Flow cytometry

Fluorochrome-conjugated isotype controls, anti-human CD3 (clone HIT3a), CD4 (clone OKT4), CD8 (clone HIT8a), CD45 (clone HI30), CD45RA (clone HI100), CD62L (clone DREG56), CD107a (clone H4A3), and CD279 (PD-1, clone EH12.2H7) were purchased from BD Biosciences (Franklin Lakes, NJ) or BioLegend. CARs were detected with AF647- or AF488-conjugated goat anti-human IgG-Fc Fab-fragments (Jackson ImmunoResearch, West Grove, PA). All antibody staining was performed in PBS containing 1% FBS after blocking with PBS containing either 10% human serum (Capricorn Scientific, Ebsdorfergrund, Germany) or 20 μg/mL mouse-IgG (Sigma-Aldrich). Samples were fixed with Cell Fix (BD Biosciences) before analysis, except when 7-aminoactinomycin D (7-AAD) staining was used. For 7-AAD staining, antibody stainings were performed as described and 7-AAD (BioLegend) was added 5 min before acquisition. Flow cytometric data were acquired using an LSR II cytometer (BD Biosciences) and analyzed using FlowJo (version 10; Tree Star, Ashland, OR).

### Statistical analysis

Statistical analysis was performed using the GraphPad Prism software (GraphPad Software, Inc., La Jolla, CA). Graphs indicate mean values and standard deviation. Since the measurements performed are not expected to lead to biased results, we assumed normal distribution of the sample values around the mean. Based on that assumption, experiments comparing three or more groups in terms of one nominal factor were analyzed using one-way analysis of variance (ANOVA) and Dunnett's *post hoc* test to identify groups statistically different from the control group; experiments with two nominal factors were analyzed by two-way ANOVA and Bonferroni's *post hoc* test. Indications: *p ≤ 0.05, **p ≤ 0.01, ***p ≤ 0.001.

## Results and Discussion

### gB-CAR-T cells produced with AB recognize and kill HCMV-infected MSCs

Since gB is an antigen highly expressed on the surface of cells during HCMV reactivation, it is a promising target for engineering CAR-T cells as a potentially HLA-independent T cell therapy. Previously, others have shown that gB-CAR-T cells containing an scFv derived from a non-neutralizing antibody, incorporating the CD28 costimulatory domain, and generated through RNA transfection became activated in the presence of gB, but failed to exhibit effector functions against HCMV-infected cells.^36^ This lack of functionality has been interpreted as the consequence of HCMV-mediated T cell suppression mechanisms that abrogated gB-CAR-T cell function and cytotoxicity.^37,38^ The authors attributed this failure to antiapoptotic viral factors and identified UL37x1 and UL36 as HCMV proteins that conferred the ability of infected cells to escape lysis by T cells.^37^

**Figure 1.**
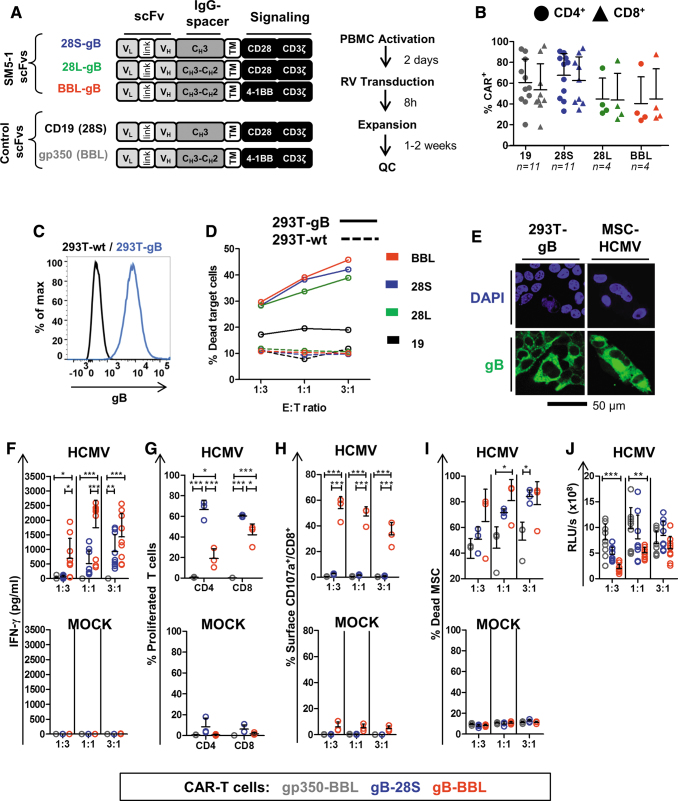
Generation of CAR-T cells with AB mononuclear cells and functionality testing. **(A)** *Left panel*: Schematic representation of CAR designs. gB-CARs incorporate scFv sequences of the SM5-1 monoclonal antibody (V_L_ → V_H_). Signaling domains are derived from CD28 (28) or 4-1BB (BB). IgG spacers are short (S: C_H_3) or long (L: C_H_3-C_H_2). Irrelevant control CARs used in the study are CD19 (28S) and gp350 (BBL). *Right panel*: Schematic representation of the production of CAR-T cells. **(B)** CAR expression shown as percentage for CD4^+^ (*circles*) or CD8^+^ (*triangles*) T cells. (19: *black*; 28S: *blue*; 28L: *green*; BBL: *red*). Results of individual production runs are shown. The numbers of runs performed for each CAR type are indicated. *Bars* represent mean values ± SD. For gating strategies, see Supplementary Figure 1A. **(C)** Flow cytometry analyses for cell surface detection of gB on a reference cell line generated after transduction with a lentiviral vector expressing gB, sorting of gB^+^ cells, and clone selection (*blue line*). 293T-wt (*black line*) shown as control. Gating is shown in Supplementary Figure S1B. **(D)** CAR-T cells (BBL: *red*; 28S: *blue*; 28L: *green*; 19: *black*) were coincubated with 293T-gB (*bold lines*) or 293T-wt (*dashed lines*) at different effector to target ratios (E:T) of 1:3; 1:1; and 3:1. Dead 293T cells were detected after 48 h of coculture by flow cytometry (7-AAD^+^ CD4^−^/CD8^−^ cells). Gating strategies are shown in Supplementary Figure 1C. **(E)** Confocal microscopy analyses showing the cell nuclei (*blue*, DAPI staining) and gB expression (*green*) in 293T-gB cells and in MSCs infected with HCMV (3 dpi). **(F–J)** CAR-T cells (gp350: *gray*; 28S: *blue*; BBL: *red*) generated with AB mononuclear cells were cocultured with MSCs infected with HCMV/GLuc (HCMV) or noninfected (MOCK, *lower panels*). Cells were cocultured for 3 days at E:T ratios of 1:3; 1:1; and 3:1. **(F)** Supernatants were collected after coculture, and the concentration of IFN-γ in the medium was measured by ELISA. **(G)** CD4^+^ and CD8^+^ T cell proliferation quantified for the 3:1 (E:T) cocultures as the percentage of cells showing loss of the labeling dye CellTrace. **(H)** Degranulation was measured by detection of the CD107a cell surface marker on CD8^+^ T cells by flow cytometry analyses. **(I)** Dead MSCs were quantified as frequency of CD45^−^ cells incorporating the viability dye eFluor450. **(J)** Detection of bioluminescence in coculture supernatants, which was correlated with the persistence of HCMV/GLuc infection in MSCs. Data shown as RLUs. Results were obtained with three independent experiments with triplicate cocultures for each experiment using CAR-T cells generated from three different HCMV-seronegative adult donor leukaphereses are shown. Triplicate cultures were pooled for flow cytometry analyses shown in **(G)**, **(H)**, and **(I)**. See Supplementary Figure S3 for detailed gating strategies. Statistical differences were evaluated by two-way ANOVA and Bonferroni's *post hoc* test. *Symbols* represent individual replicates and *bars* represent mean ± SD. For detailed statistical analysis see Table 1. *p ≤ 0.05, **p ≤ 0.01, ***p ≤ 0.001. 7-AAD, 7-aminoactinomycin D; AB, adult blood; ANOVA, analysis of variance; CAR, chimeric antigen receptor; DAPI, 4′,6-diamidino-2-phenylindole; dpi, days postinfection; ELISA, enzyme-linked immunosorbent assay; gB, glycoprotein B; GLuc, *Gaussia* luciferase; HCMV, human cytomegalovirus; IFN, interferon; IgG, immunoglobulin G; MSC, mesenchymal stem cell; PBMC, peripheral blood mononuclear cells; QC, quality control; RLUs, relative light units; RV, retroviral vector; scFvs, single-chain variable fragments; SD, standard deviation; V_H_, variable heavy; V_L_, variable light. Color images are available online.

To re-evaluate the concept of gB-CAR-T cells, we developed novel vectors and a novel *in vitro* MSC/HCMV infection model to systematically test and compare the potency of our novel-generation gB-CAR-T cells with costimulatory endodomains derived from CD28 or 4-1BB genes. Furthermore, we evaluated whether a novel scFv based on the more recently described human highly neutralizing and high-affinity SM5-1 antibody^24–26^ would prove more effective for gB-CAR-T cell engineering. Codon-optimized V_L_ and V_H_ DNA sequences of the anti-gB antibody SM5-1 were used to generate the scFv. Retroviral vectors expressing second-generation CAR variants were created by incorporating the CD28-signaling domain and a short (C_H_3) IgG-C_H_ spacer (28S), or the CD28-signaling domain and a long (C_H_2-C_H_3) IgG-C_H_ spacer (28L), or with a 4-1BB (CD137)-signaling domain with the long spacer (BBL) (Fig. 1 A). The V_L_ → V_H_ orientation for the scFv design showed better expression in pilot studies (data not shown) and these vectors were chosen for further studies. As irrelevant control CARs, we used a CD19-CAR incorporating an scFv against CD19 and the CD28 signaling domain (abbreviated to “19,” and used as control for 28-gB-CAR) and a gp350-CAR incorporating an scFv against the EBV antigen gp350 and the 4-1BB signaling domain (abbreviated to “gp350,” and used as control for the BBL-gB-CAR). CAR-T cells from AB were generated by retroviral transduction after 2 days of activation on αCD3/CD28-coated plates in the presence of cytokines. Analyses of CAR expression on the surface of CD4^+^ and CD8^+^ T cells were performed by flow cytometry 4 days after transduction and the cells were further expanded for 1 to 2 weeks (Fig. 1 A). CD4^+^ and CD8^+^ T cells expressing CARs with short IgG spacers (19 and 28S) showed ∼60% CAR positivity, whereas CARs with longer spacers (28L and BBL) showed ∼40% CAR positivity (Fig. 1B, see gating strategy in Supplementary Fig. S1A, left panel). Analyses of the relative fluorescence intensity indicated moderately higher levels of CAR expression by the BBL-gB-CAR-T cells compared with the 28-gB-CAR-T cells (not statistically significant; Supplementary Fig. S1A, right panel).

The capability of the gB-CAR-T cells to specifically detect gB and subsequently become activated was initially tested with a stably transduced clonal 293T cell line expressing gB (Fig. 1C; see gating strategy in Supplementary Fig. S1B). Forty-eight-hour cocultures of AB-generated CAR-T cells with 293T-wt or 293T-gB at different effector to target ratios (E:T 1:3, 1:1, and 3:1) were performed (Fig. 1D; see gating strategy in Supplementary Fig. S1C). Irrelevant CD19-CAR-T cells were used as control. Death of target cells was evaluated by 7-AAD staining gated on CD4^−^/CD8^−^ cells using flow cytometry. Approximately 10% nonspecific 293T-wt killing was detectable for this assay for all CAR-T cell modalities, attributable to allo-activation via TCRs reactive against allo-antigens presented by the 293T. CD19-CAR-T cells produced ∼15% dead 293T-gB cells, independently of the E:T ratios. On the contrary, when 293T-gB target cells were cultured with the different types of gB-CAR-T cells, ∼30% to 40% of the target cells died and cytotoxicity was directly correlated with the E:T ratio, confirming recognition of the gB antigen on the cell surface by the gB-CAR-T cells.

To test if the different novel modalities of gB-CAR-T cells could *bona fide* kill cells after HCMV infection without the confounding factor of allo-reactivity, we established novel assays using CB-derived MSCs. MSCs are broadly available primary cells and, since they express very low levels of HLAs, they stimulate very low allo-responses. MSCs infected with HCMV/GLuc at broad ranges of MOI (10^−4^–10^2^) showed a sigmoid curve correlating with the bioluminescent signal resultant from the reporter gene GLuc and detectable by luminometry (Supplementary Fig. S2A). MSCs infected with HCMV/GLuc at an MOI of 3 × 10^−2^, washed, and cultured for 3 days maintained ∼20% cell viability within the first 3–8 dpi while showing high expression of gB (Fig. 1E). Infection was further confirmed by flow cytometry analyses of intracellular IE-1 detection (Supplementary Fig. S2B) and detection of gB on the cell surface (Supplementary Fig. S2C).

After establishing the MSC-based HCMV infection model, we compared the potency of 28S- and BBL-gB-CAR-T cells using as irrelevant control gp350-CAR-T cells. All CAR-T cells were generated in parallel from three HCMV seronegative AB donors and were then cocultured with MSCs infected with HCMV/GLuc or MOCK-infected MSCs. All cocultures were set up and analyzed in triplicate, except for flow cytometry analyses, for which the cocultures were pooled (Fig. 1F–J; for detailed statistical analyses see Table 1; see gating strategy in Supplementary Fig. S3). The levels of secreted IFN-γ were at E:T 1:1 and 3:1, significantly higher for BBL-gB-CAR-T cells compared with 28S-gB-CAR-T cells after coculture with infected MSCs, whereas at similar conditions, no IFN-γ was detectable in cocultures with gp350-CAR-T cells (Fig. 1F). The fold proliferation of 28S-gB-CAR-T cells was significantly greater than for BBL-gB-CAR-T cells (Fig. 1G), but a significantly higher potency of BBL-gB-CAR-T cells compared with 28S-gB-CAR-T cells was observed for the degranulation assays (*i.e.*, frequency of CD8^+^ CAR-T cells expressing CD107a on the cell surface) (Fig. 1H) and for detection of dead infected MSCs (Fig. 1H). Finally, a bioluminescence signal detectable in the coculture of gp350-CAR-T cells with infected MSCs was significantly higher than the levels detectable for the cocultures with BBL-gB-CAR-T cells, directly indicating eradication of infected cells in this coculture experiment (Fig. 1J). In summary, we concluded both BBL- and 28S-gB-CAR-T cells were specifically activated, expanded, and killed HCMV-infected MSCs. Furthermore, the data indicated a better overall antitarget cytotoxic performance for BBL-gB-CAR-T cells.

**Table 1.** Comparisons of 28S-gB-CAR-T, BBL-gB-CAR-T, and irrelevant control gp350-CAR-T cells after a single coculture with mesenchymal stem cells infected with human cytomegalovirus

	28S	BBL	gp350	p		
	Mean (SD)	Mean (SD)	Mean (SD)	28S × BBL	28S × gp350	BBL × gp350
AB (*n* = 3)						
IFN-γ (pg/mL)	9.3E+02 (5.78E+02)	1.43E+03 (8.0E+02)	0 (0)	ns	^**^	^***^
% CD107a^+^/CD8^+^	7.97E-01 (4.1E-01)	3.3E+01 (9.5E+00)	3.23E-01 (2.03E-01)	^***^	ns	^***^
% Dead MSC	8.4E+01 (5.25E+00)	7.77E+01 (1.79E+01)	5.0E+01 (1.41E+01)	ns	^*^	ns
% Proliferated T cells (CD4^+^)	6.65E+01 (9.09E+00)	1.9E+01 (9.57E+00)	6.87E-01 (3.7E-01)	^***^	^***^	^*^
% Proliferated T cells (CD8^+^)	6.02E+01 (1.04E+00)	4.21E+01 (1.05E+01)	2.4E-01 (2.6E-02)	^*^	^***^	^***^
CB (*n* = 3)						
IFN-γ (pg/mL)	4.96 + 00 (9.90E+00)	1.84E+02 (1.56E+02)	5.52E+00 (7.95E+00)	^***^	ns	^***^
% CD107a^+^/CD8^+^	3.65E+00 (1.06E+00)	1.02E+01 (1.10E+00)	1.97E+00 (2.65E-01)	^***^	ns	^***^
% Dead MSC	1.62E+01 (1.47E+00)	2.87E+01 (7.11E+00)	1.63E+01 (2.66E+00)	^***^	ns	^***^
% Proliferated T cells (CD4^+^)	4.31E+00 (2.00E+00)	3.59E+00 (2.17E+00)	1.16E+00 (3.5E-01)	ns	^**^	^*^
% Proliferated T cells (CD8^+^)	9.72E+00 (2.38E+00)	8.33E+00 (2.8E+00)	3.66E+00 (1.71E+00)	ns	ns	ns

CAR-T cells were generated from three AB donors and from three CB donors and cultured with MSC-HCMV or MSC-MOCK at E:T 1:3; 1:1; and 3:1 for 72 h. Values are shown for 3:1 cocultures of CAR-T cells with MSC/HCMV. Triplicate cultures for each donor were analyzed individually for IFN-γ and bioluminescence detection. Cultures were pooled for flow cytometry analyses. Statistical significance was determined by two-way ANOVA and Bonferroni's *post hoc* test between the indicated groups and *p*-values are shown.

^*^p ≤ 0.05, ^**^p ≤ 0.01, ^***^p ≤ 0.001.

AB, adult blood; ANOVA, analysis of variance; CAR, chimeric antigen receptor; CB, cord blood; gB, glycoprotein B; HCMV, human cytomegalovirus; IFN, interferon; MSC, mesenchymal stem cell; ns, not significant (*p* > 0.05); SD, standard deviation.

### Potency of AB-gB-CAR-T cells after sequential cocultures with MSC-HCMV

**Figure 2.**
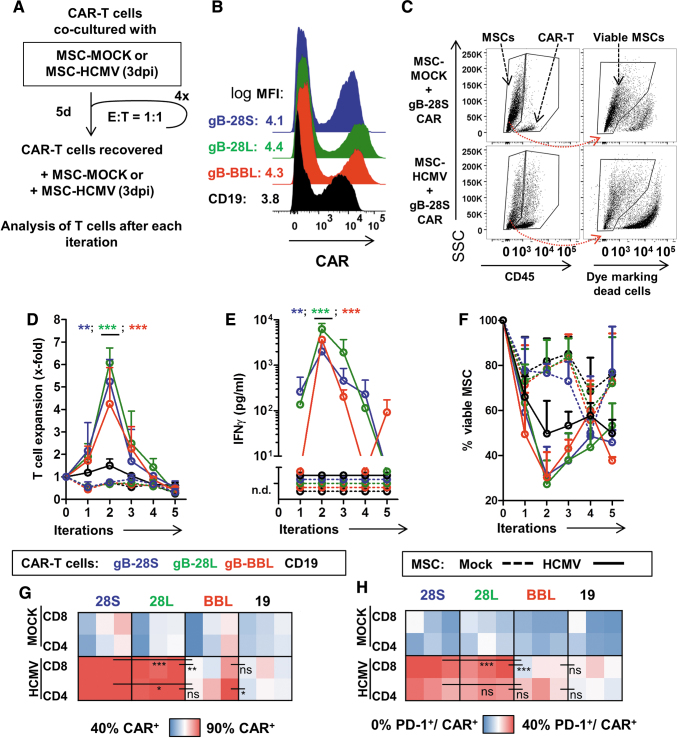
Effects of sequential cocultures of CAR-T cells with MSCs infected with HCMV. **(A)** Schematic illustration of the sequential coculture assay. CAR-T cells were cocultured with MSCs noninfected (MOCK) or infected with HCMV/GLuc (HCMV) at E:T 1:1 for 5 days. The expanded T cells were recovered, counted, and reincubated with fresh target cells for additional four cycles. Triplicate experiments were performed using one AB T cell source for generation of CAR-T cells, and MSCs expanded from three different donors. **(B)** Flow cytometric detection of CAR^+^ T cells (19: *black*; 28S: *blue*; 28L: *green*; BBL: *red*). **(C)** Flow cytometry approach to distinguish CD45^+^ T cells and CD45^−^ MSCs and then staining with the viability dye FVD eFluor450 to detect viable MSCs. Representative examples show cocultures of 28S CAR-T cells with MSCs. **(D)** Expansion of the CAR-T cells of different types upon culture with MSCs was determined by flow cytometry using counting beads as reference. *Asterisks* indicate differences between cocultures with HCMV-infected MSCs comparing gB-CARs (indicated by *asterisk color*) to CD19-CAR by two-way ANOVA and Bonferroni's *post hoc* test. **(E)** Coculture supernatants were analyzed by ELISA for detection of IFN-γ. **(F)** Quantification of viable MSCs after iterations. **(D–F)** *Dots* represent mean ± SD (*n* = 3 measured for each MSC source). **(G)** Heat map representing frequency of CAR^+^ cells measured for CD4^+^ or CD8^+^ T cells after the last iteration (lowest 40% *blue*, to highest 90% *red*). Each *square* depicts one experimental replicate (*n* = 3 in total per coculture). **(H)** Heat map representing frequency of PD-1^+^ on CAR^+^/CD4^+^ or CAR^+^/CD8^+^ T cells (lowest 0% *blue*, to highest 40% *red*). Differences were analyzed by two-way ANOVA and Bonferroni's *post hoc* test. **(D–H)** *p ≤ 0.05, **p ≤ 0.01, ***p ≤ 0.001. For detailed statistical analysis see Table 2. See Supplementary Figure S4 for gating strategies. MFI, mean fluorescence intensity; ns, not significant (*p* > 0.05). Color images are available online.

After showing that both BBL- and 28S-gB-CAR-T cells were capable of killing infected MSCs after a single exposure to the target, we evaluated if sequential exposures of the gB-CAR-T cells to HCMV-infected MSCs would also result into serial target detection and killing. A sequential killing assay was established using CAR-T cells cultured with MSC-MOCK or with MSC-HCMV at E:T ratios of 1:1 for 5 days. Then, nonadherent T cells were recovered from the cocultures, a fraction was analyzed, and a second fraction was recultured at the initial E:T ratio with fresh infected MSCs for additional four iterations. This assay was performed with CAR-T cells generated with AB from a single donor and in three independent experiments using three different MSC target sources to confirm reproducibility (Fig. 2A; for detailed statistical analyses see Table 2). 28S-, 28L-, and BBL-gB-CAR-T and 19-CAR-T cells were produced in parallel and 19-CAR T cells were used as an irrelevant control (Fig. 2B). For this sequential killing assay, the frequencies of the remaining viable MSCs were determined by excluding the cells incorporating the viability dye (Fig. 2C). Using two measures of T cell activation (expansion and secretion of IFN-γ), we observed the initial expansion of all gB-CAR-T cells in the presence of MSC-HCMV, but not with MSC-MOCK (Fig. 2D). T cell expansion in cocultures with MSC-HCMV reached a peak level at iteration 2 and all gB-CAR-T cells showed a significantly greater expansion than CD19-CAR-T cells (Table 2). Expansion dropped afterward, which could be attributed to a putative T cell exhaustion. For IFN-γ-secretion, significant differences were consistently found between gB-CAR-T cells and CD19-CAR-T cells (Fig. 2E; Table 2), and a modestly higher IFN-γ secretion for T cells expressing the 28L-gB-CAR compared with the two other gB-CAR constructs for the first 3 iterations. These results indicate that, first, the CD28-endodomain leads to stronger activation of T cells compared with the 4-1BB domain, and second, the long C_H_3-C_H_2 spacer slightly outperformed the short C_H_3 spacer construct when comparing the two CD28-endodomain containing constructs. Regarding reduction of viable MSC-HCMV cells, for all tested gB-CAR constructs, the lowest frequency of detectable viable infected cells was reached at the second coculture (Fig. 2F). There was a background cytotoxicity seen with 19-CAR-T cells, which could be attributed to allo-reactivity. After the last iteration, statistical analysis revealed no significant differences in the viability of MSC-HCMV exposed to all the gB-CAR-constructs (compared with the 19-CAR). These results indicated that, despite their differences in T cell activation, all gB-CAR-constructs tested showed long-lasting cytotoxic effects against HCMV-infected cells. The surface expression of the CAR and PD-1 was analyzed with the available remaining cells after the last fifth cycle of coculture with the HCMV-infected MSCs (Fig. 2G; see gating strategy in Supplementary Fig. S4). Although at the beginning of the cocultures the frequencies of CAR^+^ T cells were similar for all CAR-constructs (∼40%; Fig. 2B), after the fifth iteration, we observed a positive selection for 28S- and 28L-gB-CAR-T cells (>90% of T cells became CAR^+^; Fig. 2G). On the contrary, BBL-gB-CAR-T cell cultures after the fifth iteration were more heterogeneous and 50–90% of the T cells expressed the CAR (Fig. 2G). CAR^+^ T cell frequencies were significantly lower for 19- or BBL-gB-CAR-T cells cultured with MSC-HCMV compared with 28S- or 28L-gB-CAR-T cells (Table 2). The frequencies of CAR-T cells expressing the activation/exhaustion marker PD-1 after repeated exposure to MSC-HCMV were dependent on the type of CAR^+^ T cells, thus significantly higher for the 28S- and 28L-gB-CAR-T cells when compared with the BBL-gB-CAR-T cells (Fig. 2H; Table 2). In sum, these results confirmed that all versions of the gB-CAR-T cells generated with AB killed MSCs infected with HCMV effectively up to two iterations, but upon chronic re-exposure to MSC-HCMV, the performance dropped. After five iterations, the 28S and 28L constructs maintained the CAR expression but also showed significantly higher CAR^+^/PD-1^+^ T cell frequencies than the BBL construct. Corroborating observations by others,^39^ this would imply that the CAR-T cells incorporating the 4-1BB-derived endodomain had a weaker tonic CAR signaling but were less prone to exhaustion.

**Table 2.** Comparisons of 28S-gB-CAR-T, BBL-gB-CAR-T, and irrelevant control CD19-CAR-T cells after five sequential cocultures with mesenchymal stem cells/human cytomegalovirus

	28S	28L	BBL	CD19	p					
	Mean (SD)	Mean (SD)	Mean (SD)	Mean (SD)	28S × 28L	28S × BBL	28S × CD19	28L × BBL	28L × CD19	BBL × CD19
After second iteration										
Number of T cells	5.24E+05 (9.80E+04)	6.08E+05 (6.63E+04)	4.25E+05 (1.62E+05)	1.50E+05 (2.87E+04)	ns	ns	^***^	^*^	^***^	^***^
IFN-γ (pg/mL)	2.02E+03 (1.12E+03)	6.24E+03 (2.01E+03)	3.65E+03 (5.98E+02)	0 (0)	^***^	^*^	^**^	^***^	^***^	^***^
% Viable MSC	3.12E+01 (1.27E+01)	2.73E+01 (1.06E+01)	3.03E+01 (1.13E+01)	4.97E+01 (1.46E+01)	ns	ns	ns	ns	ns	ns
After last iteration										
% CAR^+^/CD4^+^	9.28E+01 (3.2E-01)	9.18E+01 (2.31E+00)	8.23E+01 (8.07E+00)	7.08E+01 (4.01E+00)	ns	^*^	^***^	ns	^***^	^*^
% CAR^+^/CD8^+^	9.45E+01 (8.0E-01)	8.97E+01 (1.28E+00)	7.52E+01 (8.16E+00)	7.22E+01 (5.05E+00)	ns	^***^	^***^	^**^	^***^	ns
% PD-1^+^/CAR^+^/CD4^+^	2.80E+01 (6.66E+00)	2.61E+01 (2.92E+00)	1.81E+01 (7.98E+00)	3.76E+00 (7.0E-01)	ns	ns	^***^	ns	^***^	^*^
% PD-1^+^/CAR^+^/CD8^+^	4.18E+01 (7.49E+00)	3.48E+01 (5.81E+00)	6.59E+00 (3.09E+00)	8.56E+00 (5.31E+00)	ns	^***^	^***^	^***^	^***^	ns

The following groups were compared: 28S-gB-CAR-T, 28L-gB-CAR-T, BBL-gB-CAR-T, and the irrelevant reference control CD19-CAR-T cells. CAR-T cells were generated from a single AB donor. Cocultures were performed in independent triplicate experiments, and for each experiment, MSCs derived from a different donor were used. Results pertaining to T cell numbers, IFN-γ detection, and remaining viable MSCs are shown for cocultures of CAR-T cells with MSC/HCMV after the second iterations. Results pertaining to the frequencies of CD4^+^ and CD8^+^ CAR-T cells and PD-1^+^CD4^+^ CAR-T and PD-1^+^CD8^+^CAR-T are shown for the cells recovered after the last iteration. Statistical significance was determined by two-way ANOVA and Bonferroni's *post hoc* tests between the indicated groups.

^*^ *p* ≤ 0.05, ^**^p ≤ 0.01, ^***^p ≤ 0.001.

### CB-derived BBL-gB-CAR-T cells reacted against and killed MSC-HCMV more efficiently than 28S-gB-CAR-T cells

**Figure 3.**
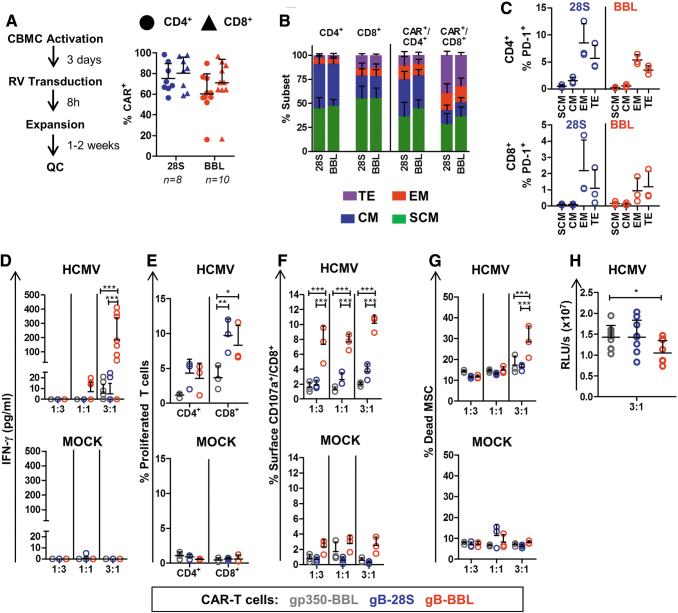
Generation of CAR-T cells with CB cells and functionality testing. **(A)** *Left panel*: Schematic representation of CAR-T cell production using CB units. After transduction of mononuclear cells with vectors expressing 28S- or BBL-CAR, the frequencies of CD4^+^ CAR^+^ (*circles*) and CD8^+^ CAR^+^ (*triangles*) T cells were analyzed. The numbers of transduction runs are indicated, *symbols* represent each run and *bars* represent mean ± SD (see Supplementary Fig. S1 A for gating strategy). **(B)** 28S- and BBL-gB-CAR-T cells were produced using three different CB donations. The T cell subtypes for total CD4^+^ or CD8^+^ CAR^+^ T cells and CD4^+^ CAR^+^ and CD8^+^ CAR^+^ T cells were quantified by flow cytometry (Gating strategy: Supplementary Fig. S5): TE (CD45RA^+^/CD62L, *purple*), EM (CD45RA^−^/CD62L^−^, *red*), CM (CD45RA^−^/CD62L^+^, *blue*), SCM (CD45RA^+^/CD62L^+^, *green*). **(C)** Frequencies of PD-1^+^ cells detectable for CD4^+^ or CD8^+^ T cells for each subtype for 28S- (*blue*) or BBL-CAR-T cells (*red*). **(D–H)** CAR-T cells (gp350: *gray*; 28S: *blue*; BBL: *red*) generated with CB mononuclear cells were cocultured with MSCs infected with HCMV/GLuc (HCMV) or noninfected (MOCK, *lower panels*). **(D)** Cell were cocultured for 3 days at E:T ratios of 1:3; 1:1; and 3:1. Supernatants were collected after coculture and the concentration of IFN-γ in the medium was measured by ELISA. **(E)** CD4^+^ and CD8^+^ T cell proliferation quantified for the 3:1 (E:T) cocultures as the percentage of cells showing loss of the labeling dye CellTrace. **(F)** Degranulation was detected by detection of the CD107a cell surface marker on CD8^+^ T cells by flow cytometry analyses. **(G)** Dead MSCs were quantified as frequency of CD45^−^ cells incorporating the viability dye eFluor450. **(H)** Detection of bioluminescence in coculture supernatants (E:T 3:1), which was correlated with the persistence of HCMV/GLuc infection in MSCs. Data shown as RLUs. Results obtained with three independent experiments with triplicate cocultures for each experiment using CAR-T cells generated from three different CB units are shown. Triplicate cultures were pooled for flow cytometry analyses shown in **(E–G)**. See Supplementary Figure S3 for detailed gating strategies. Statistical differences were evaluated by two-way ANOVA and Bonferroni's *post hoc* test **(D–G**) or by one-way ANOVA and Dunnett's *post hoc* test **(H)**. *Symbols* represent individual replicates and *bars* represent mean ± SD. For detailed statistical analysis see Table 1. *p ≤ 0.05, **p ≤ 0.01, ***p ≤ 0.001. CB, cord blood; CM, central memory; EM, effector memory; SCM, stem cell memory; TE, terminal effector. Color images are available online.

Since T cells from CB are in general more quiescent than T cells from AB, CB CD34^−^ fractions were activated with beads (αCD2/CD3/CD28) and cytokines for 3 days before transduction. Beads and cytokines were also used for the post-transduction expansion (Fig. 3A, left panel). Using these methods, we obtained ∼70% transduction efficiency for CD4^+^ and 60% for CD8^+^ 28S- and BBL-gB-CAR-T cells (Fig. 3A). For further functional characterizations, the two types of gB-CAR-T cells were generated in parallel using CB from three different donors. The transduction efficiencies and the immune phenotypic features did not differ much between the gB-CAR-T cell types, except for higher frequencies of terminal effector T cells in CD8^+^ 28S-gB-CAR-T cells compared with CD8^+^ BBL-gB-CAR-T cells (*p* = 0.066) (Fig. 3B; see gating strategy in Supplementary Fig. S5). Expression of PD-1 was observed mostly in effector memory and terminal effector gB-CAR-T cells, and was higher in 28S-expressing T cells, possibly due to higher tonic CAR signaling (Fig. 3C).

CAR-T cells (28S-, BBL, and the irrelevant gp350 control) were cocultured with MSC-HCMV or MSC-MOCK (all cocultures were set up in triplicate, which were pooled for FACS analyses; for detailed statistical analyses see Table 1). The levels of secreted IFN-γ after coculture with infected MSCs were significantly higher for BBL- compared with 28S-gB-CAR-T cells, whereas IFN-γ was nondetectable upon cocultures of CAR-T cells with MSC-MOCK (Fig. 3D; Table 1). Although coculture with MSC-HCMV promoted a modestly higher expansion for 28S- compared with BBL-gB-CAR-T cells (Fig. 3E), the frequencies of CD8^+^ CAR-T cells expressing CD107a on the cell surface and frequencies of dead infected MSCs promoted by cytotoxicity were significantly higher for the BBL-gB-CAR-T cells (Fig. 3F, G; Table 1). In addition, significantly lower levels of bioluminescence (generated by HCMV/GLuc spread in MSCs) were detectable in cocultures with BBL- and 28S-gB-CAR-T cells compared with the control gp350-CAR-T cells (Fig. 3H). Therefore, we confirmed for CB-derived cells that BBL-gB-CAR-T cells showed the more favorable antiviral functional properties than 28S-gB-CAR-T cells as measured by IFN-γ secretion, degranulation, and cytolysis of HCMV-infected MSCs.

### BBL-gB-CAR-T cells tested in a humanized mouse model of HCMV infection

**Figure 4.**
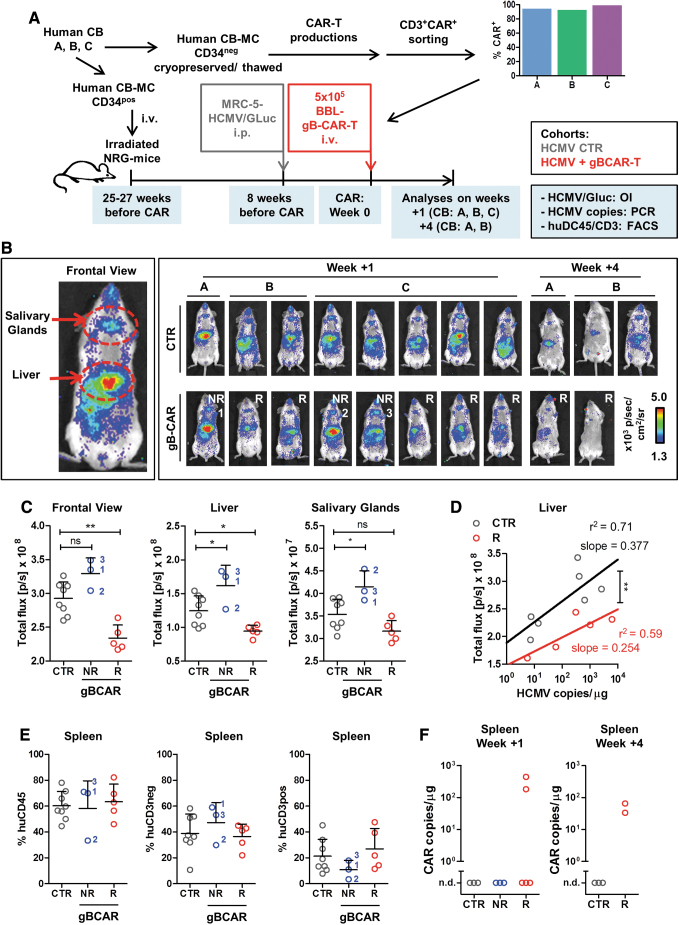
Therapeutic efficacy of BBL-gB-CAR-T cells in humanized mice infected with HCMV. **(A)** Schematic representation of humanized mouse model: Irradiated NRG mice were transplanted with CB CD34^+^ hematopoietic stem cells from three different CB donors (CB: A–C). CD34^−^ fractions were cryopreserved. Seventeen weeks after transplantation, once humanized mice reached a high human immune reconstitution, they were challenged i.p. with MRC-5 fibroblasts infected with HCMV/GLuc. BBL-gB-CAR-T cells were then produced with the autologous thawed CD34^−^ cells. CD3^+^/CAR^+^T cells were sorted, shortly expanded, and flow cytometry analyses were performed showing >90% CAR positivity. Eight weeks after HCMV infection, one cohort was treated with 5 × 10^5^ BBL-gB-CAR-T cells injected i.v. and the other control cohort was not treated (CTR). Final analyses were performed 1 week (CTR *n* = 8; gB-CAR *n* = 8) or 4 weeks after CAR-T cell administration (CTR *n* = 3; gB-CAR *n* = 2). **(B)** Descriptive OI analyses to follow HCMV/GLuc infection levels and biodistribution. *Left panel*: Representative example of the frontal view showing that HCMV/GLuc infection was evident predominantly in the anatomical regions of salivary glands and liver, and also spreading toward other regions of the body. *Right panel*: Imaging results for all mice used in the studies (CB reconstitutions: A–C, CTR or CAR-treated, terminal analyses at weeks 1 or 4 after treatment). The quantified bioluminescence was expressed as p/sec/cm^2^/sr, pseudo-*color dots* represent lower signals in *blue* and higher in *red*. Mice treated with CAR-T cells and showing lower bioluminescence signal intensities relative to controls were classified as responders (R; *n* = 5 of 8) and the others nonresponders (NR; *n* = 3 of 8). **(C)** To determine the HCMV infection levels noninvasively, the OI signals were analyzed by photon quantification. Graphs show values obtained by applying same-sized ROIs for all mice set as frontal view, liver or salivary glands. Results for each mouse are shown for controls (CTR; *gray*), CAR-T-treated nonresponders (NR; *blue*), and CAR-T-treated responders (R; *red*). *Bars* represent mean ± SD. Statistical differences were analyzed by one-way ANOVA and Dunnett's *post hoc* test by comparison of the treated cohorts with the control cohort. **(D)** RT-qPCR analyses to determine HCMV copies were performed with DNA obtained from leukocytes isolated from livers from each mouse. Shown in the *x*-axis are the data obtained for mice analyzed at week +1. Shown in the *y*-axis are the data for the equivalent mice regarding OI-signals measured in the liver-ROI. The correlations were analyzed by linear regression. The correlation curves of control mice (*black* line. r^2^ = 0.71, slope = 0.377) and CAR-T cell-treated responder mice (*red* line. r^2^ = 0.59, slope = 0.254) differed significantly in elevation (analysis of covariance, ANCOVA; *p* = 0.0046). Data obtained from nonresponders did not differ from control mice (not shown). **(E)** Human immune reconstitution measured in the spleen of mice 1 week after CAR-T cell treatment. Shown are the percentages of human CD45^+^ cells in all lymphocytes (*left*), huCD45^+^/CD3^negative^ (*middle*), and huCD45^+^/CD3^positive^ (*right*). Results for each mouse are shown for controls (CTR; *gray*), CAR-T-treated nonresponders (NR; *blue*), and CAR-T-treated responders (R; *red*). *Bars* indicate mean ± SD. A one-way ANOVA analysis showed no significant differences between the cohorts, but NR mice showed higher frequencies of huCD45^+^ and huCD45^+^/CD3^negative^ cells. **(F)** DNA isolated from total spleen specimens was analyzed for detection of integrated CAR-copies by RT-qPCR. gB-CAR sequences were detectable only in responder mice of the CAR-T cell-treated cohort transplanted with CB donor B (*n* = 4 of 7 responders overall). For detailed statistical analysis see Table 3. *p ≤ 0.05; **p ≤ 0.01; ***p ≤ 0.001. i.p., intraperitoneal; i.v., intravenous; NRG, Nod.Rag.Gamma; OI, optical imaging; ROIs, regions of interest; RT-qPCR, quantitative real-time PCR. Color images are available online.

We used our recently described HCMV/GLuc infection model to determine whether BBL-gB-CAR-T cells could lower HCMV infection *in vivo*. This *in vivo* model is based on long-term (17 weeks) humanized NRG mice transplanted with human CB CD34^+^ HSCs and injected i.p. with MRC-5 fibroblasts infected with HCMV/GLuc. Noninvasive analyses by optical imaging enabled visualization and quantitative measurements of HCMV reactivations and spread particularly in the anatomical regions of the liver and salivary glands.^27^ We have previously shown in this model that the long-term human myeloid and stem cell reconstitution enabled HCMV-infection and the mice acquired human functional HCMV-reactive T and B cell responses.^27^ Here, we used CB CD34^+^ cells to transplant the mice and the CB CD34^−^ fraction was used to generate the “autologous” BBL-gB-CAR-T cells (Fig. 4A). Humanized mice reconstituted with three different CB units (“A,” “B,” and “C”) showed long-term human immune reconstitutions for 17 weeks with ∼50% human CD45^+^ cells in blood (data not shown). CD3^+^CAR^+^ BBL-gB-CAR-T cells generated with these CB units were sorted by FACS and, after expansion, >90% of the CD4^+^ and CD8^+^ cells were CAR^+^ (Fig. 4A). Intravenous gB-CAR-T cell administration was performed 8 weeks after HCMV infection. Subgroups of mice were analyzed 1 week (untreated controls, CTR *n* = 8; gB-CAR *n* = 8) or 4 weeks (CTR *n* = 3; gB-CAR *n* = 2) after gB-CAR-T administration. HCMV/GLuc infection and spread were evaluated by optical imaging for ROIs as the frontal view or anatomical regions of liver and salivary glands. Whereas control mice showed consistent levels of bioluminescence, mice treated with gB-CAR-T cells showed a bimodal distribution: five out of eight mice showed significantly lower levels of bioluminescence signals relative to controls, and they were considered “responders” (“R,” Fig. 4C; Table 3). The remaining three out of eight mice showed, on the contrary, increased bioluminescence signals, compared with the CTR group and were designated “nonresponders” (“NR”; Fig. 4C; Table 3). RT-qPCR for analyses of HCMV-genomic copies in DNA isolated from single-cell suspensions of the liver was performed. The results were plotted in the *x*-axis against the quantified values obtained for optical imaging analyses in the *y*-axis (Fig. 4D). The linear regression analyses comparing the CTR and gB-CAR-T cell “responders” showed a significantly higher optical imaging signal, inferring higher infection in the CTR group (∼30% to 40% higher regression curve elevations), while retaining the relationship of HCMV-copies in DNA with the optical imaging signal (the regression curve slopes did not differ). Although the huCD45 frequencies in the spleen were similar for all the groups, responder mice showed a noticeable increase in the frequencies of CD3^positive^ cells in the spleen (Fig. 4E), corroborating the hypothesis of a CAR-T cell-mediated viral control. Hitherto, CAR detection by PCR was possible for only two out of the eight mice in the gB-CAR-T cell responder group, analyzed 1 week after therapy (Fig. 4F). For the analyses performed 4 weeks after gB-CAR-T cell administration, CAR expression was detectable in two out of two analyzed mice (Fig. 4F). Analyses by flow cytometry showed low levels of CAR expression on CD4^+^ T cells recovered from bone marrow (Supplementary Fig. S6B), but not from other tissues. Only one mouse showed detectable CD8^+^ gB-CAR-T cells in the liver 1 week after treatment (data not shown). The CAR-T cell-treated mice showed no adverse clinical symptoms (loss of body weight, observable change in behavior, eczema, and GvHD). In summary, gB-CAR-T cell administration into humanized mice infected with HCMV resulted into a subset of responders. The response was correlated with higher levels of T cells in the spleen and, occasionally, with CAR detection in the bone marrow and spleen.

**Table 3.** Comparisons between BBL-gB-CAR-T cell-treated relative to the control nontreated humanized mice infected with human cytomegalovirus/Gaussia luciferase

1 Week		CTR	NR	R	p	
		Mean (SD)	Mean (SD)	Mean (SD)	CTR × NR	CTR × R
OI: Full abdomen	Pixels/s	2.92E+08 (2.26E+07)	3.29E+08 (1.91E+07)	2.34E+08 (1.73E+07)	ns	^**^
OI: Liver		1.25E+08 (2.04E+07)	1.61E+08 (2.47E+07)	9.46E+07 (7.76E+06)	^*^	^*^
OI: Salivary glands		3.54E+07 (3.09E+06)	4.15E+07 (2.90E+06)	3.16E+07 (2.10E+06)	^*^	ns
RT-qPCR: Liver	Copies/μg	5.04E+02 (8.22E+02)	6.93E+02 (4.89E+02)	1.51E+03 (2.35E+03)	ns	ns

Optical imaging analyses quantified in regions of interest on the full abdomen, the liver region, and the salivary gland regions, as well as RT-qPCR detection of HCMV-copies in the liver. Shown are results for individuals analyzed 1 week after administration of the T cells. The control group was not treated with CAR-T cells (CTR); the CAR-T cell-treated group was divided into responder (R) and nonresponder individuals (NR). Statistical significance was determined by one-way ANOVA and Dunnett's *post hoc* tests comparing with the control group.

^*^p ≤ 0.05, ^**^p ≤ 0.01, ^***^p ≤ 0.001.

OI, optical imaging; RT-qPCR, quantitative real-time PCR.

## Conclusions: Affinity-Engineering of gB-CAR-T Cells

*In vitro* potency assays conclusively showed that both BBL- and 28S-gB-CAR-T cells produced with AB or CB recognized and reacted against HCMV infection. These assays could exclude that allo-reactivity was a confounding factor, since: (1) MSCs express low levels of HLAs; (2) the irrelevant CAR-T cells showed low to no background against allogeneic infected MSCs; and (3) we used only HCMV-seronegative AB or CB donors to avoid possible anti-HCMV memory responses mediated against infected cells by TCRs. Thus, exploring an scFv derived from the high-affinity gB-antibody SM5-1 for CAR design enabled a high on-target effect lowering a potential off-target toxicity. This “affinity engineering” is a well-known concept applied for the development of T cells expressing transgenic TCRs.^40^

Previously, other groups showed that gB-CAR-T cells containing an scFv derived from a non-neutralizing lower affinity antibody, incorporating the CD28 costimulatory domain and generated through RNA transfection, became activated in the presence of gB, but failed to exhibit effector functions against HCMV-infected cells.^36^ This lack of functionality has been interpreted as the consequence of HCMV-mediated T cell suppression mechanisms that abrogated gB-CAR-T cell function and cytotoxicity.^37,38^ The authors attributed this failure to antiapoptotic viral factors and identified UL37x1 and UL36 as HCMV-proteins that confer the ability of infected cells to escape lysis by T cells.^37^ Although this is an interesting hypothesis, the described mechanisms would also apply to HCMV-specific T cells targeting infected cells via the native TCR, but it known that HCMV-reactive T cells indeed control virus reactivations in HSCT recipients.^10^ Thus, a general blockade of gB-CAR-T cell cytotoxic effector functions by HCMV-infected cells through antiapoptotic UL37x1 and UL36 viral factors seems an unlikely explanation. Rather, possible reasons for the lack of success of the previous gB-CAR-T engineering attempts may be the lower affinity scFv used or a transient expression of the CAR after RNA transfection.

We also compared the effects of different signaling domains in gB-CAR-T cells. It has been described that CARs targeted to CD19 expressed from γ-retroviral vectors and harboring the 4-1BB costimulatory domain showed lower T cell exhaustion induced by antigen-independent tonic signaling than CAR-T cells harboring the CD28 costimulatory domain.^41^ Here, we observed that the expression of PD-1 was lower for gB-CAR-T cells harboring the 4-1BB costimulatory domains. This is important since upon chronic antigen activation, PD-1 is upregulated on CD8^+^ T cells, which can induce further upregulation of its ligand PD-L1 on target cells. The binding of the ligand PD-L1 to PD-1 results in T cell apoptosis. Indeed, it has been suggested that highly activated CD8^+^PD-1^+^ cytotoxic T lymphocytes (CTLs) can have a negative impact on adoptive memory CTLs used for HCMV immune cell therapy.^12^ Correspondingly, CB-gB-CAR-T cells harboring 4-1BB also showed significantly higher IFN-γ secretion, degranulation activity, and cytotoxicity against HCMV-infected MSCs than gB-CAR-T cells with the CD28 costimulatory domain. 28S-gB-CAR-T cells, on the contrary, showed a higher proliferation potential, which may explain why they were more prone to exhaustion.

The main objective of the *in vivo* potency testing recommended by the European Medicine Agency is to show that the engineered cells can perform their supposed clinical attribute(s). Therefore, our HCMV/GLuc infection model in fully humanized mice was chosen as a relevant preclinical model. We observed a partial antiviral response, which was associated with a moderate increase of T cell frequency. Since CAR-T cells are “living drugs,” mice with a human immune system are quite useful to predict the function and interaction of CAR-T cells with the other “natural” players of the immune system. Although still with limitations, humanized mouse models are thus becoming accepted and important to predict and even remediate adverse effects of CAR-T cells such as neurotoxicity,^42^ cytokine release syndrome,^42–44^ and off-target effects. Although gB-CAR-T cells will undoubtedly require further technological optimizations (such as further improved signaling domains, combinatorial third-generation CD28/4-1BB CARs or coexpression of cytokines), the antiviral specificity and potency of gB-CAR-T cells *in vitro* are now proven and the *in vivo* data with BBL-gB-CAR-T cells also indicate a therapeutic effect.

Taken as a whole, CAR-T cell technologies as antiviral therapies are continuously evolving in regard to optimization of scFvs, signaling designs, and stability of expression of the CAR transgene.^45–47^ Here, engineering of CAR-T cells with an scFv design based on a high-affinity neutralizing anti-gB antibody fused to 4-1BB showed conclusive results for the control of HCMV infection *in vitro* and encouraging results *in vivo*. A therapeutic variability could be expected and still remains to be minimized by new CAR delivery approaches, such as by gene editing to abrogate the TCR signaling.

For clinical translation, we can envisage that a fraction of the apheresis of the HCMV-negative stem cell donors or of the CB unit could be used for production of donor-matched gB-CAR-T cells. The cells could be used prophylactically or could be cryopreserved for administration upon signs of HCMV reactivation. With new technologies in hand such as gene editing to knock out the TCR and HLA expression, gB-CAR-T cells can also be produced as “third-party” and offer a novel “off-the-shelf” prospect for treating patients with HCMV reactivations.

## Supplementary Material

Supplemental data

Supplemental data

Supplemental data

Supplemental data

Supplemental data

Supplemental data
