# Supplementary figures and images for: Adult and Cord Blood-Derived High-Affinity gB-CAR-T Cells Effectively React Against Human Cytomegalovirus Infections
Source: Hum Gene Ther. 2020 Apr 16;31(7-8):423–39. doi: 10.1089/hum.2019.149 (PMC7194322; doi:10.1089/hum.2019.149)

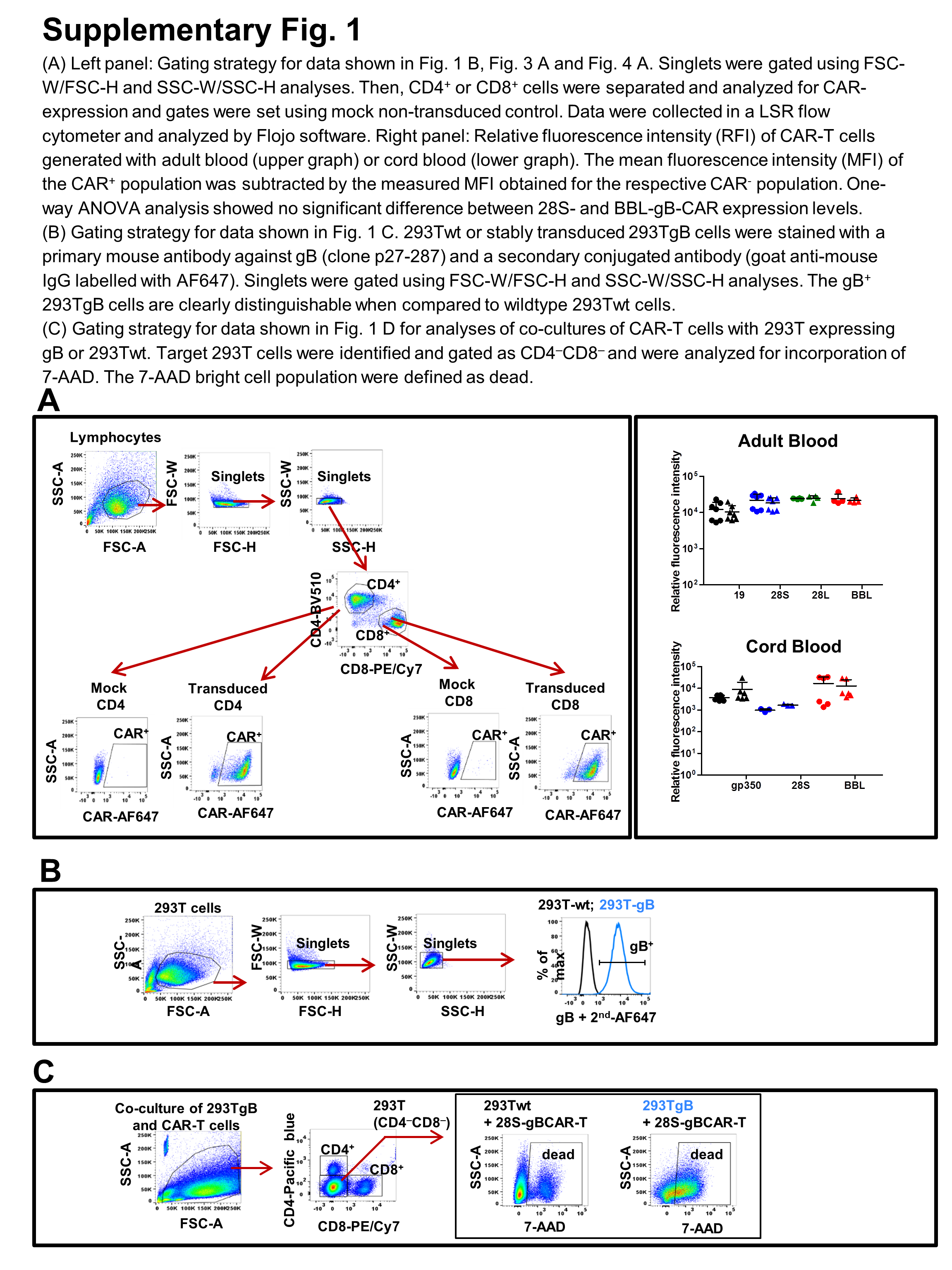

Supplement: Supplemental data [file Supp_Fig_S1.tif]

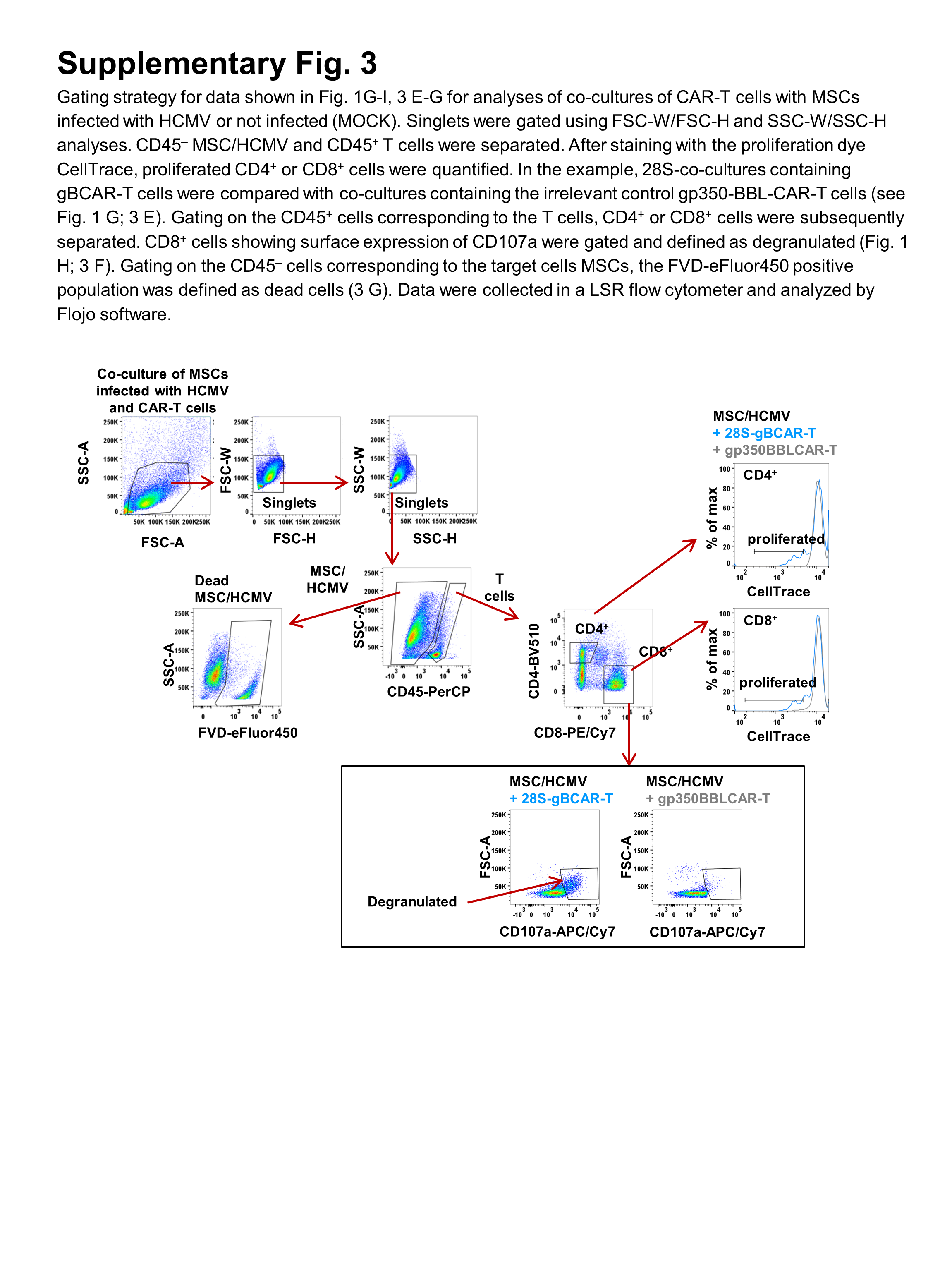

Supplement: Supplemental data [file Supp_Fig_S3.tif]

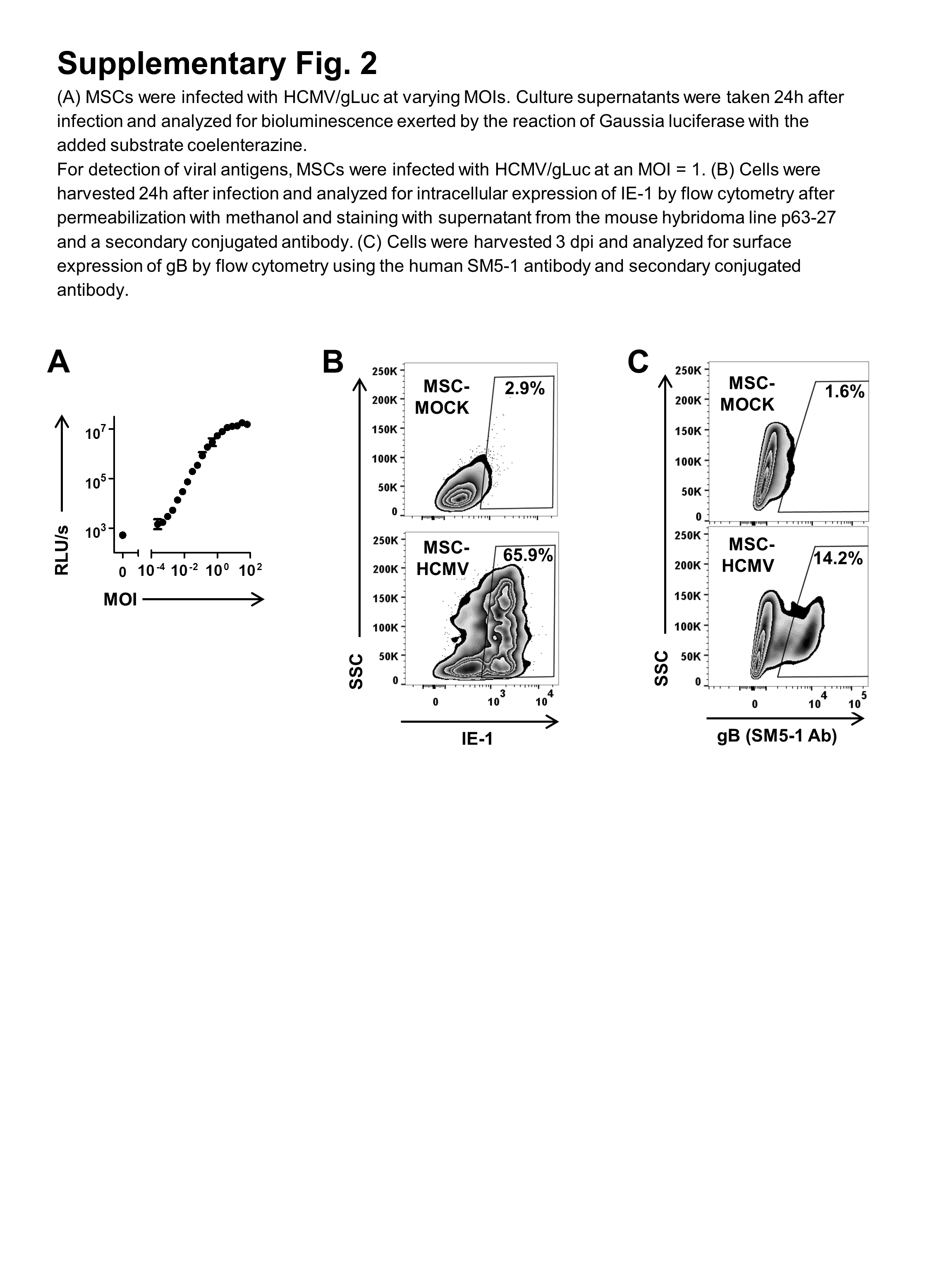

Supplement: Supplemental data [file Supp_Fig_S2.tif]

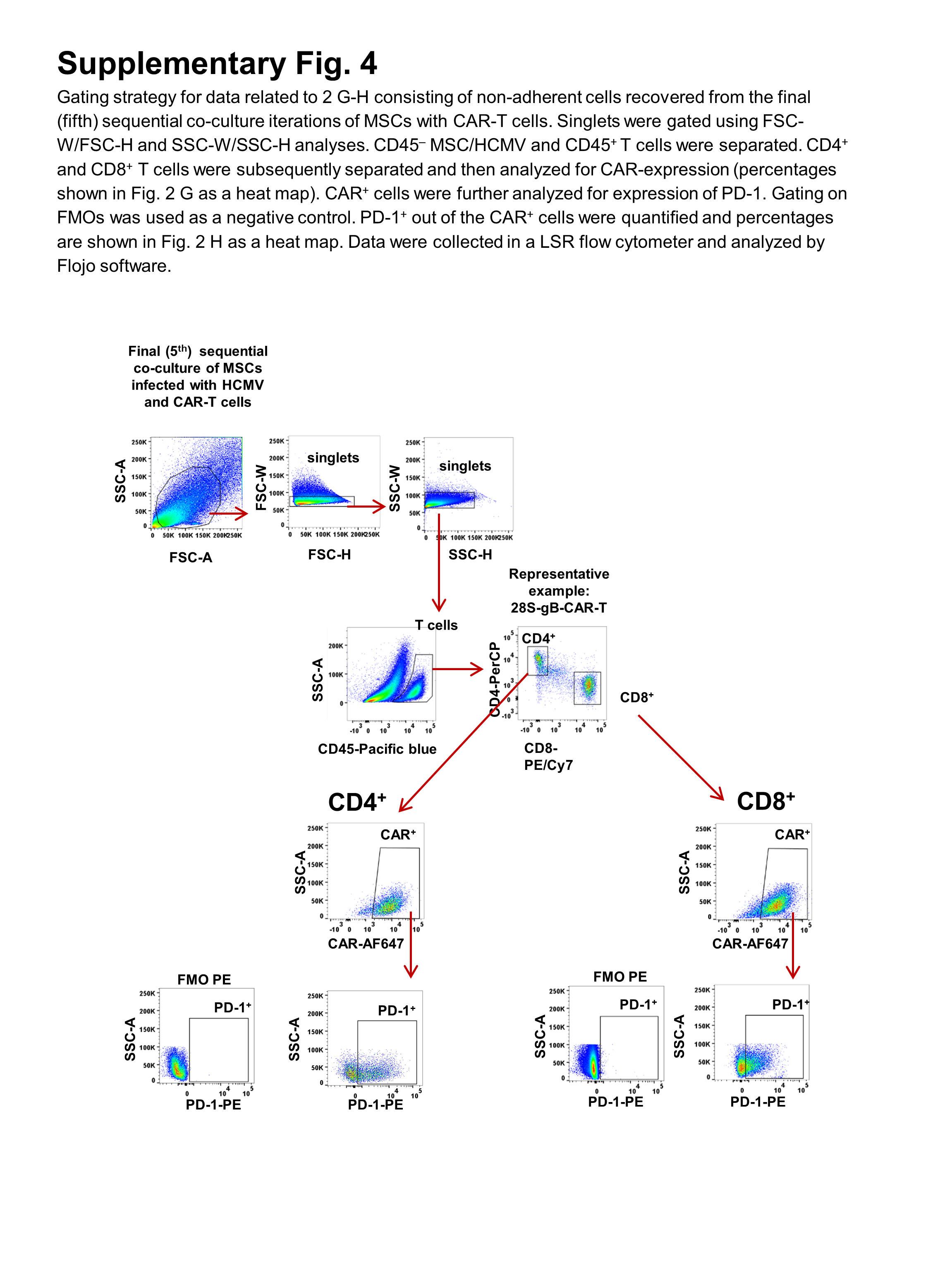

Supplement: Supplemental data [file Supp_Fig_S4.tif]

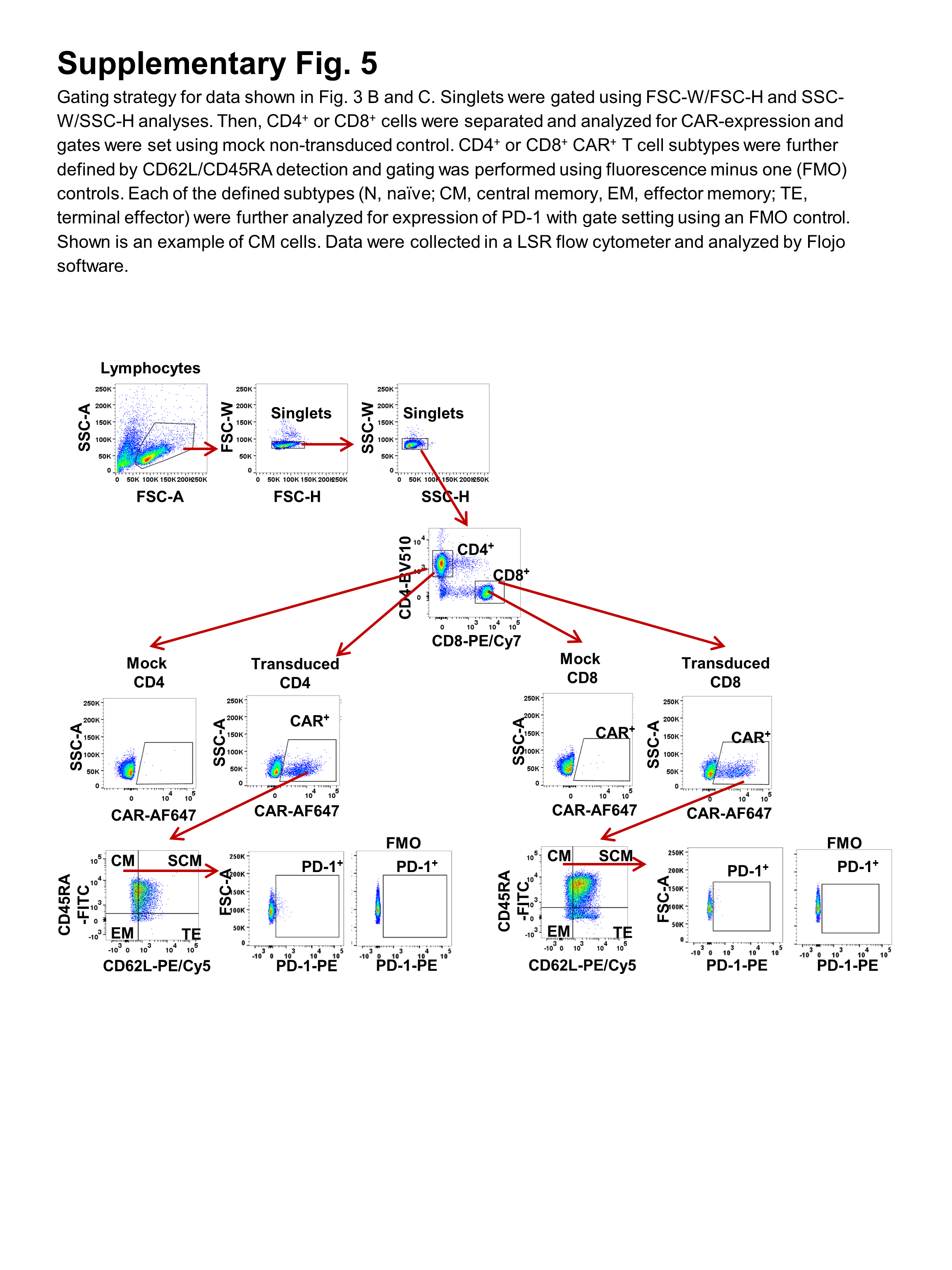

Supplement: Supplemental data [file Supp_Fig_S5.tif]

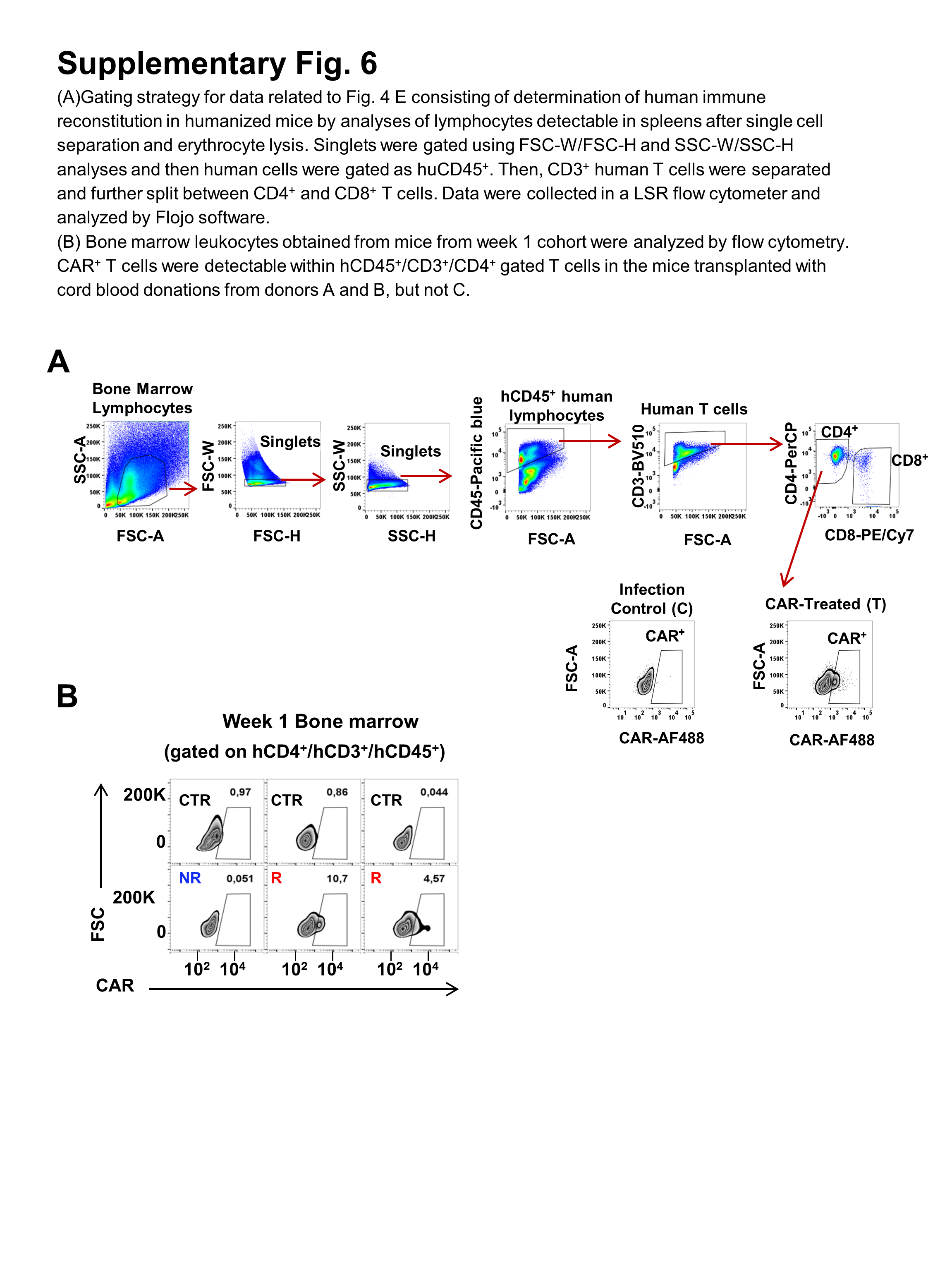

Supplement: Supplemental data [file Supp_Fig_S6.tif]
